# Shielding from UV Photodamage: Implications for Surficial Origins of Life Chemistry on the Early Earth

**DOI:** 10.1021/acsearthspacechem.0c00270

**Published:** 2021-01-29

**Authors:** Zoe R. Todd, Jack W. Szostak, Dimitar D. Sasselov

**Affiliations:** † Center for Astrophysics Harvard and Smithsonian, 60 Garden Street, Cambridge, Massachusetts 02138, United States; ‡ Howard Hughes Medical Institute, Department of Molecular Biology and Center for Computational and Integrative Biology, Massachusetts General Hospital, Boston, Massachusetts 02114, United States

**Keywords:** prebiotic chemistry, UV photochemistry, early earth, origins of life, shielding

## Abstract

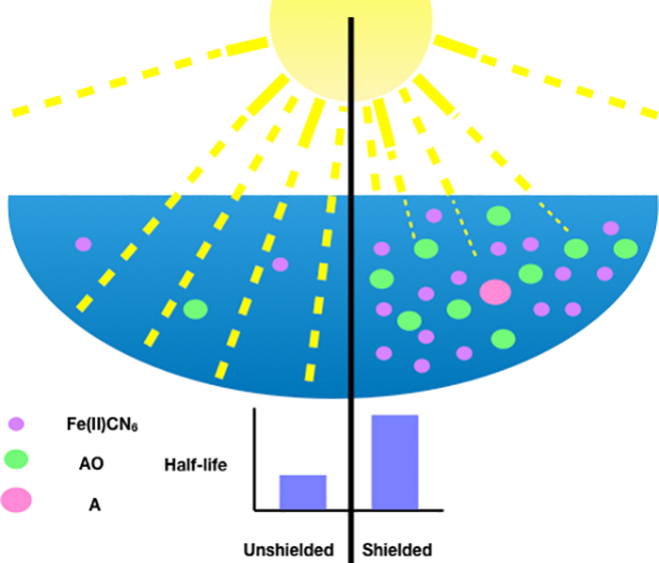

UV light has been invoked as a source of energy for driving prebiotic chemistry, but such high energy photons are also known to cause damage to biomolecules and their precursors. One potential mechanism for increasing the lifetime of UV-photounstable molecules is to invoke a protection or shielding mechanism. UV shielding could either occur by the molecule in question itself (self-shielding) or by the presence of other UV-absorbing molecules. We investigate and illustrate these two shielding mechanisms as means of increasing the lifetime of 2-aminooxazole (AO), a prebiotic precursor molecule moderately susceptible to UV photodamage, with an expected half-life of 7 h on the surface of the early Earth. AO can be protected by being present in high concentrations, such that it self-shields. AO can similarly be protected by the presence of UV-absorbing nucleosides; the degree of protection depends on the concentration and identity of the nucleoside. The purine nucleosides (A, G, and I) confer more protection than the pyrimidines (C and U). We find that 0.1 mM purine ribonucleosides affords AO about the same protection as 1 mM AO self-shielding, corresponding to a lifetime enhancement of 2–3×. This suggests that only a modest yield of nucleosides can potentially allow for protection of UV photounstable molecules, and therefore this could be a plausible mechanism for protecting sensitive molecules while prebiotic synthesis is occurring simultaneously. Our findings suggest that both synthetic and degradative reactions can proceed at the same time, given various degrees of shielding.

## Introduction

The role of UV light in prebiotic chemistry and the origins of life on Earth has been considered in depth as UV light can both destroy organic molecules^1,2^ and drive prebiotic processes including the synthesis of amino acids,^3^ ribonucleotides,^4^ and a wide cyanosulfidic network capable of synthesis all four major types of biomolecules.^5,6^ UV light would have been more intense on the early Earth than at present, given the larger fractional output of the young Sun in the UV^7,8^ and the lack of the UV-absorbing oxygen and ozone gases responsible for screening out UV radiation in the modern atmosphere.^9^ The energy available from UV light on the early Earth is comparable or greater than that from lightning and atmospheric shocks, making the UV-driven synthesis of building blocks of life potentially very significant.^10,11^ Ranjan and Sasselov^12^ find that UV light down to 204 nm would have been present on the early Earth, given a N_2_–CO_2_-dominated atmosphere.

UV light is known to harm biomolecules: nucleotides can undergo photodamage either as individual monomers (e.g., the pyrimidine bases form the photohydrates 6-hydroxy-5,6-dihydrocytidine/uridine^13,14^) or as part of oligonucleotides. Adjacent pyrimidine bases in a strand of RNA or DNA will form cyclobutane pyrimidine dimers or 6-4 photoproducts.^15^ Proteins, especially those with aromatic UV-absorbing residues, can also be excited by UV light, potentially damaging the protein structure.^16^ It is also interesting to note that the nucleobases used by life today show shorter excited-state lifetimes than many other closely related nucleobases after UV excitation, giving them enhanced photostability and perhaps explaining their selection for use in early life.^17^

It therefore becomes important to contrast the helpful and harmful effects of UV light for building up a prebiotic chemical inventory for the origins of life. Even if UV light is not invoked for prebiotic synthetic purposes and instead other sources of energy (e.g., lightning discharge, impact delivery/synthesis) are used, any origins of life scenario occurring on the surface of the planet will have to assess the compatibility of the invoked chemistry with the presence of these high energy photons.

All surface prebiotic chemistry need not be UV-photostable; indeed, various theories for UV protection or mitigation have been postulated in the past. Sagan^1^ suggested that early life could have been shielded from the damaging effects of UV light by the presence of layers of UV-absorbing purine and pyrimidine nucleotides. Prebiotic organic polymers (e.g., from HCN^18^), inorganic species (e.g., Cl^–^, Br^–^, Mg^2+^, SH^–^, Fe^2+^)^2,18^ or dissolved organic carbon^2^ have all been suggested to have the potential for UV-shielding effects. The accumulation of longer oligonucleotides over monomers (which are favored by hydrolysis) has also been suggested to be due to UV selection, where the nitrogenous bases absorb the UV light to protect the sugar-phosphate backbone from UV-induced cleavage.^19,20^ Furthermore, even if UV damage does occur, various mechanisms could repair these lesions, e.g., charge transfer states from decay of photoexcited DNA can repair DNA photolesions.^21,22^ Life today uses many means to repair UV damage, including photolyases and excision repair enzymes, but these would not have been available for the origin of life.

**Figure 1:**
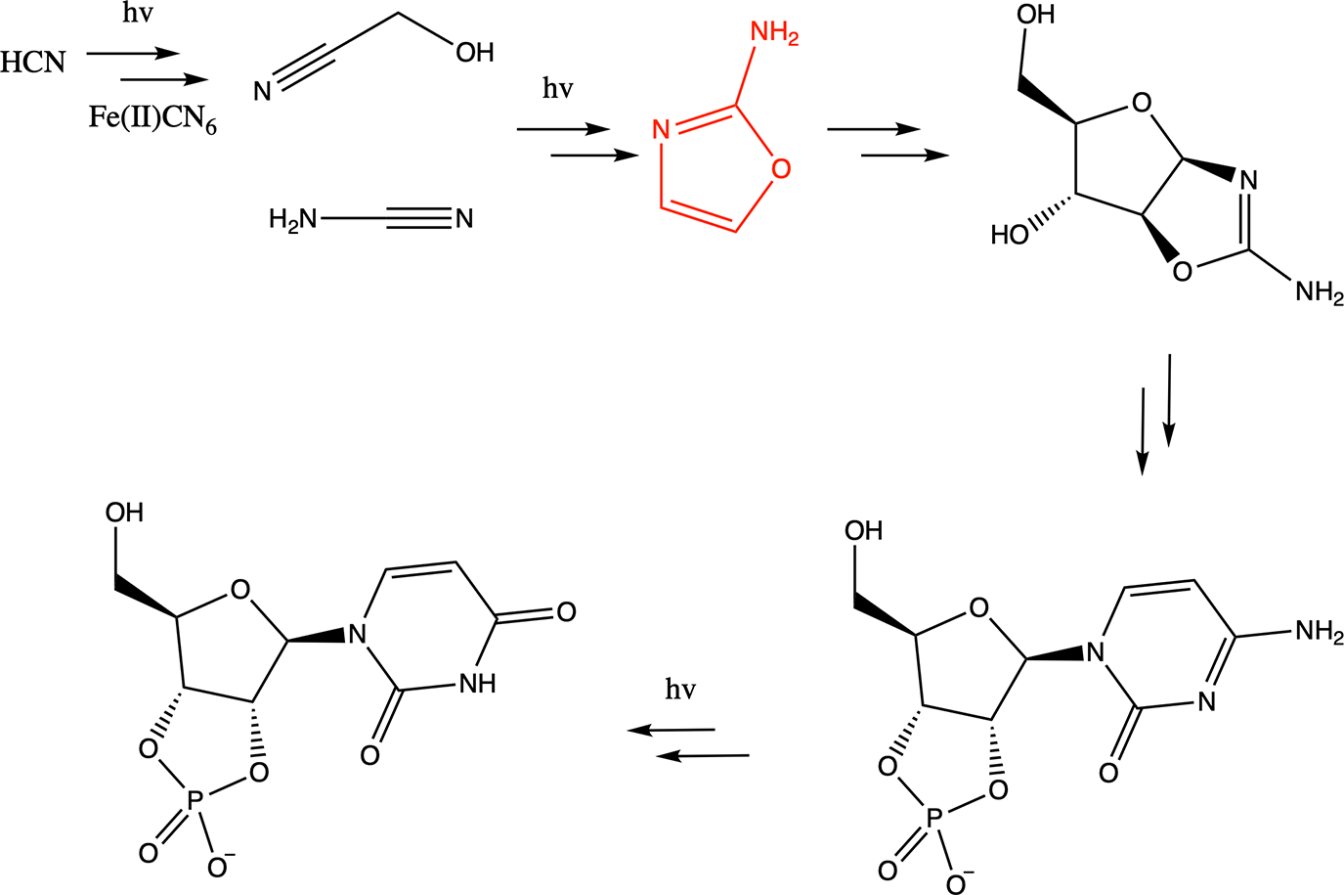
Highlighted reactions during the synthesis of pyrimidine ribonucleotides,^4^ beginning from simple molecules like HCN, glycolonitrile, cyanamide to 2-aminooxazole (shown in red). AO then goes on to form pentose amino-oxazolines and ultimately β-ribocytidine-2′,3′-cyclic phosphate and β-ribouridine-2′,3′-cyclic phosphate. The use of UV is indicated where relevant.

The origin and the sustained existence of protocells, postulated primordial cells containing a genetic material capable of self-reproduction, was dependent on the production of molecular building blocks of life over planetary (geological) timescales. These building blocks were also needed in sufficient abundance in order to allow protocells enough opportunities to evolve toward a fully self-sustainable existence. In one view of how the building blocks of life could have come about prebiotically, the Sutherland lab has invoked a cyanosulfidic network making use of simple feedstock molecules and UV light. These synthetic reactions occur over many steps to yield the desired molecules, so each individual step needs to have sufficiently high yields of intermediates in the chemical network, or intermediates must accumulate in stable reservoirs of material, through precipitation (e.g., accumulation of ferrocyanide salts^23^), crystallization (e.g., ribose aminooxazoline^6,24^), etc., in order to overcome the arithmetic demon problem (see, e.g., Gilbert and Martin^25^ for relevant discussions) and lead to sufficient concentrations of the final products. We can then consider the molecules involved as part of three groups: the feedstocks, the intermediates, and the final products, e.g., ribonucleotide monomers and oligomers. A previous work on the effects of UV light on prebiotic molecules has focused on the products (e.g., Todd et al.^26^) as well as on the feedstocks (e.g., Xu et al.^27^). Here, we focus on the intermediates and use 2-aminooxazole (AO), a key intermediate in the synthesis of pyrimidine ribonucleotides^4^ (see Figure 1) as a case study.

AO could plausibly have been synthesized on the early Earth by making use of UV light^28^ or gamma radiation.^29^ We previously examined the photostability of three 2-aminoazoles (AO, 2-aminothiazole, and 2-aminoimidazole) potentially important for prebiotic chemistry and found AO to be the least photostable, with a half-life of roughly 7 h under the spectrum of light expected on the early Earth.^30^ Excited-state dynamic simulations of AO predict the possibility of photochemically induced ring opening, leading to irreversible destruction of AO.^31^ In this study, we specifically examined if (1) AO could be self-shielded from UV light, simply by being present at higher concentrations, or (2) AO could be shielded by the presence of various nucleosides. When considering the role of UV light in such prebiotic networks, the comparison between productive, UV-driven synthesis and destructive photodegradation of intermediates and products is crucial. It is therefore critical to understand the balance between the need for UV to drive synthetic chemistry and the damage caused by UV light to certain intermediates and products. Understanding the tension between these competing factors (synthesis vs degradation) is complicated by the matter of self-shielding and absorption of UV light by other molecules present in the environment. Here, we begin to address these issues by considering the UV-driven degradation rate of AO under varying concentrations and in the presence of other UV-absorbing molecules, with the aim of constraining or placing limits on the overall consistency of prebiotic networks.

## Results and Discussion

**Figure 2:**
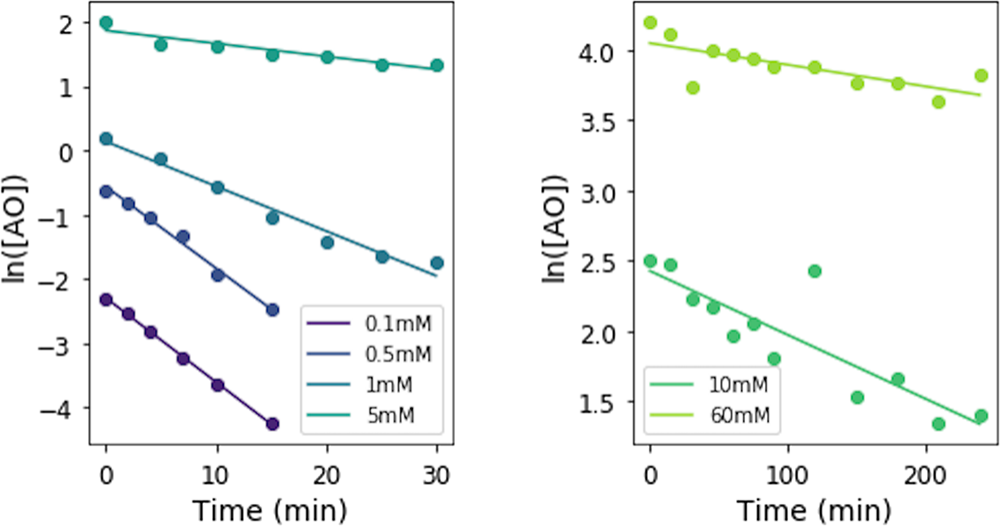
Logarithm of AO concentration (in M) as a function of irradiation time for various concentrations, as determined by UV–Vis spectroscopy. The slope of the best-fit lines represents the rate constant of the reaction.

We first examined the rate of photodestruction of AO at varying concentrations to assess the efficiency of self-shielding as a potential UV-blocking mechanism. We irradiated solutions of 0.1, 0.5, 1, 5, 10, and 60 mM AO in a Rayonet reactor (254 nm, mercury emission lamps) and determined the concentration of AO at various timepoints during the irradiation by removing an aliquot of the solution from the cuvette, diluting it, and measuring the UV–Vis absorption spectrum. The concentration of AO was calculated from a standard curve (see SI section 2). The logarithm of the concentration of AO (in M) plotted against irradiation time gives a straight line (Figure 2), whose slope represents the rate constant of AO photodestruction.

**Figure 3:**
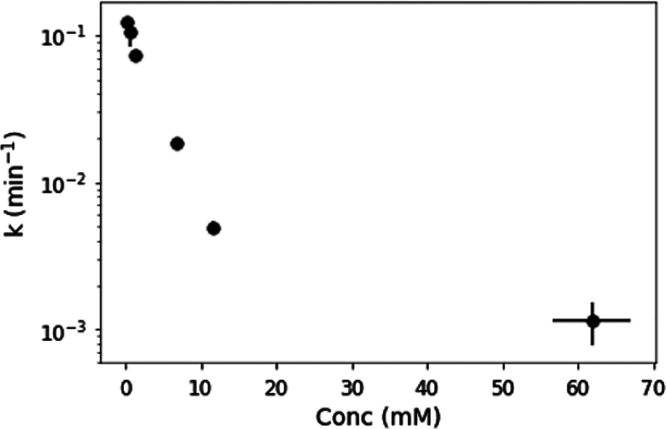
Rate constant of AO photodestruction for varying initial concentrations of AO. At higher concentrations, AO absorbs a larger amount of the UV light, leading to self-shielding and correspondingly lower rates of photodestruction. We note that the 60 mM AO point does not appear to fit the log-linear trend seen at the lower concentrations.

Figure 3 shows the rate constants for varying concentrations of AO. At higher concentrations, the transparency of the solution decreases due to increased absorption of the sample, so fewer photons can reach the entire sample, causing the rate of photodestruction to be reduced. We note that, at the concentrations of AO used in these experiments, no precipitation was observed throughout the experiments. Experiments were repeated in duplicate; points represent the average, and error bars show the standard deviation from each duplicate set. At the lowest concentration tested (0.1 mM), the rate constant is 0.12 min^–1^ under these irradiation conditions (254 nm, RPR-200 reactor). At 1 and 10 mM, the rate constant is 7.2 × 10^–2^ and 5.0 × 10^–3^ min^–1^, leading to half-life enhancements of a factor of 1.7 and 25, respectively. In Todd et al.,^30^ we found that AO (0.1 mM) has a half-life of 7 h, taking into account the wavelength dependence of the photodestruction and the spectrum of the Sun on the surface of the young Earth. The UV flux from the RPR-200 reactor is 14× greater than the flux of light expected on the early Earth from 210 to 300 nm, and the RPR-200 reactor only emits narrowband radiation centered at 254 nm. It is important to note that the experiments presented here at different concentrations are not performed in a wavelength-dependent manner and are performed at a higher flux than expected on the early Earth, and therefore we cannot determine an accurate half-life for the UV-irradiation environment present on the early Earth. However, if we assume that the lifetime enhancement of AO under 254 nm irradiation at higher fluxes is directly comparable to the lifetime enhancement under a solar-like irradiation spectrum, 1 and 10 mM solutions of AO would have half-lives of approximately 12 and 180 h on the early Earth, respectively.

In addition to investigating the lifetime enhancement of AO due to self-shielding, we also tested whether nucleosides, which are fairly UV-absorbent molecules, could enhance the half-life of AO to UV light when co-irradiated. In these experiments, AO was present in concentrations of 0.05 or 0.1 mM AO (where self-shielding is not significant) and varying concentrations (0.01, 0.05, and 0.1 mM) of nucleosides (G, C, U, A, and I) were added to these solutions. Irradiations were also carried out at 254 nm in the Rayonet reactor. The UV spectra were recorded throughout the irradiation to enable determination of the rate constants, as described below.

**Figure 4:**
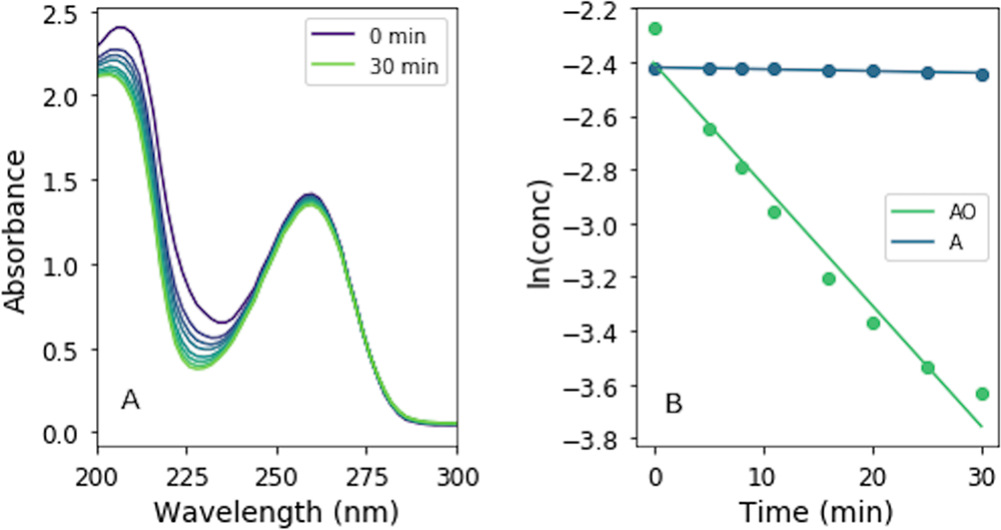
(A) Absorption spectra of a solution of 0.1 mM AO + 0.1 mM A during irradiation. The AO and A spectral features are clearly separated, and no new features appear, enabling the concentration of each species to be determined by solving eqs 1 and 2. (B) The logarithm of concentration (in M) as a function of irradiation time is used to determine the rate constants.

The purine nucleosides show a primary absorption feature around 260 nm. During irradiation, this absorption feature decreases and no new absorption signals are observed, indicating that any byproducts of AO photodegradation do not interfere with the absorption spectrum (Figure 4A). This allows for a simple extraction of the concentration of each species in solution as a function of irradiation time to enable determination of the rate constant as the slope of the best-fit line in Figure 4B. The absorption at the maximum wavelengths for AO and the purine ribonucleoside (216 and 260 nm, respectively) are used to determine the concentration by solving a system of two equations:
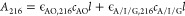
1
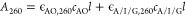
2

**Figure 5:**
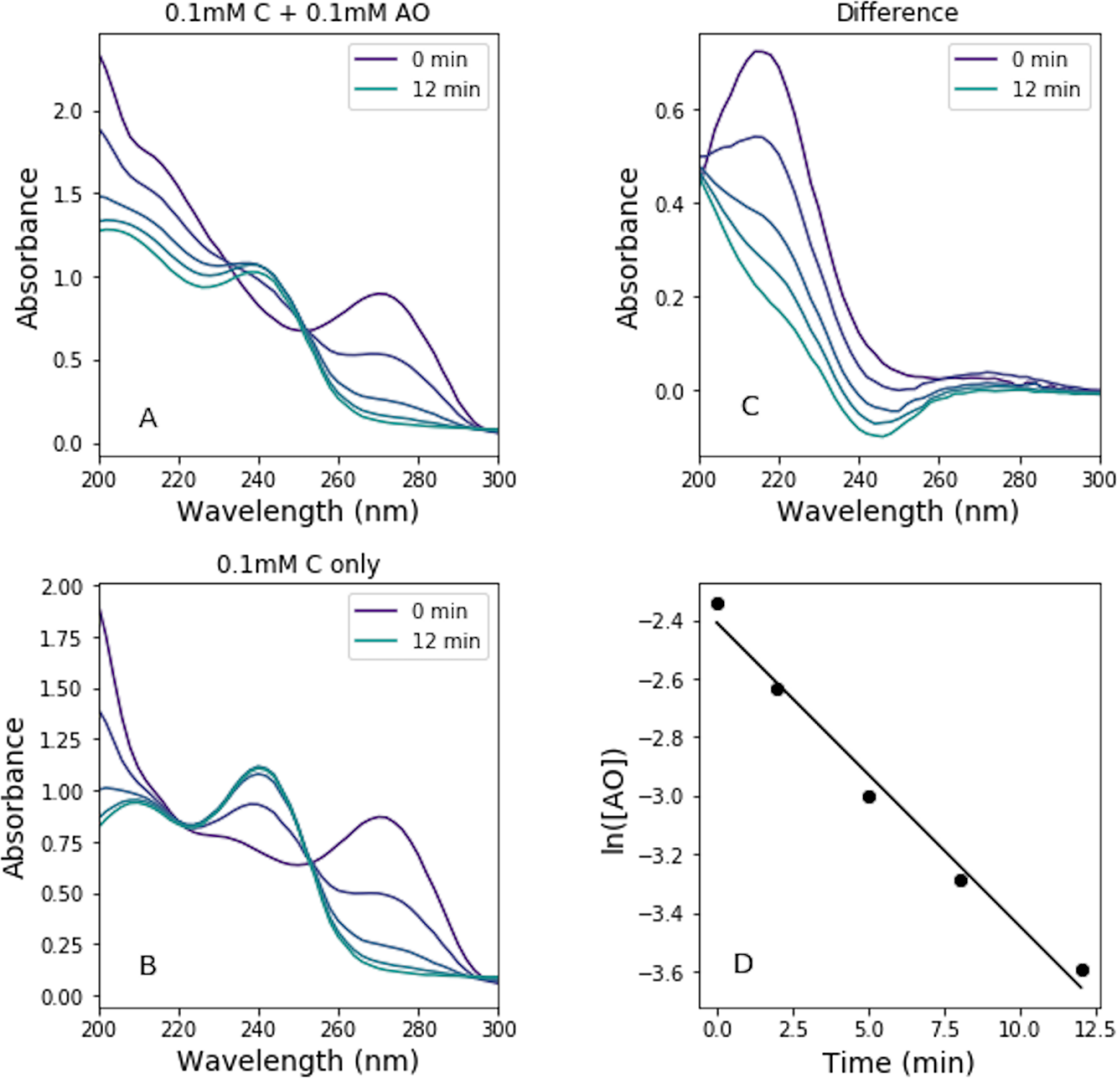
(A) Absorption spectra of a solution of 0.1 mM AO + 0.1 mM C with irradiation time. (B) Absorption spectra of a solution of 0.1 mM C with irradiation time. (C) The difference of the spectra in (A) and (B) at each respective timepoint shows the effects of irradiation on AO. (D) The logarithm of calculated effective concentration of AO (in M) with irradiation time yields an estimated rate constant for AO destruction.

This method does not work for the pyrimidine ribonucleotide experiments because the pyrimidine ribonucleosides show increasing absorption at wavelengths <240 nm during irradiation, even as the 260 nm initial feature decreases. Given the overlap of the AO band (maximum at 216 nm) and the new absorption feature that grows in with irradiation time, the extinction coefficients cannot be used to disentangle the concentrations of the pyrimidine ribonucleosides and AO as is the case for the purine ribonucleosides. Therefore, for the pyrimidine ribonucleoside experiments, identical experiments were performed with only the ribonucleoside (without the addition of AO). The spectra at each timepoint were then subtracted from the corresponding spectra of the solution of both AO and the ribonucleoside (i.e., 0.1 mM C spectra as a function of irradiation, Figure 5B, were subtracted from those of the 0.1 mM AO + 0.1 mM C solution, Figure 5A). This enabled an effective destruction rate of AO to be calculated by converting the difference spectra (Figure 5C) into the concentration of AO vs time (Figure 5D). We note that when this subtraction method is applied to the purine ribonucleoside experiments, similar rate constants are recovered as with the extinction coefficient method; however, the use of difference spectra could be subject to artifacts, so rate constants determined by this method are less certain.

**Figure 6:**
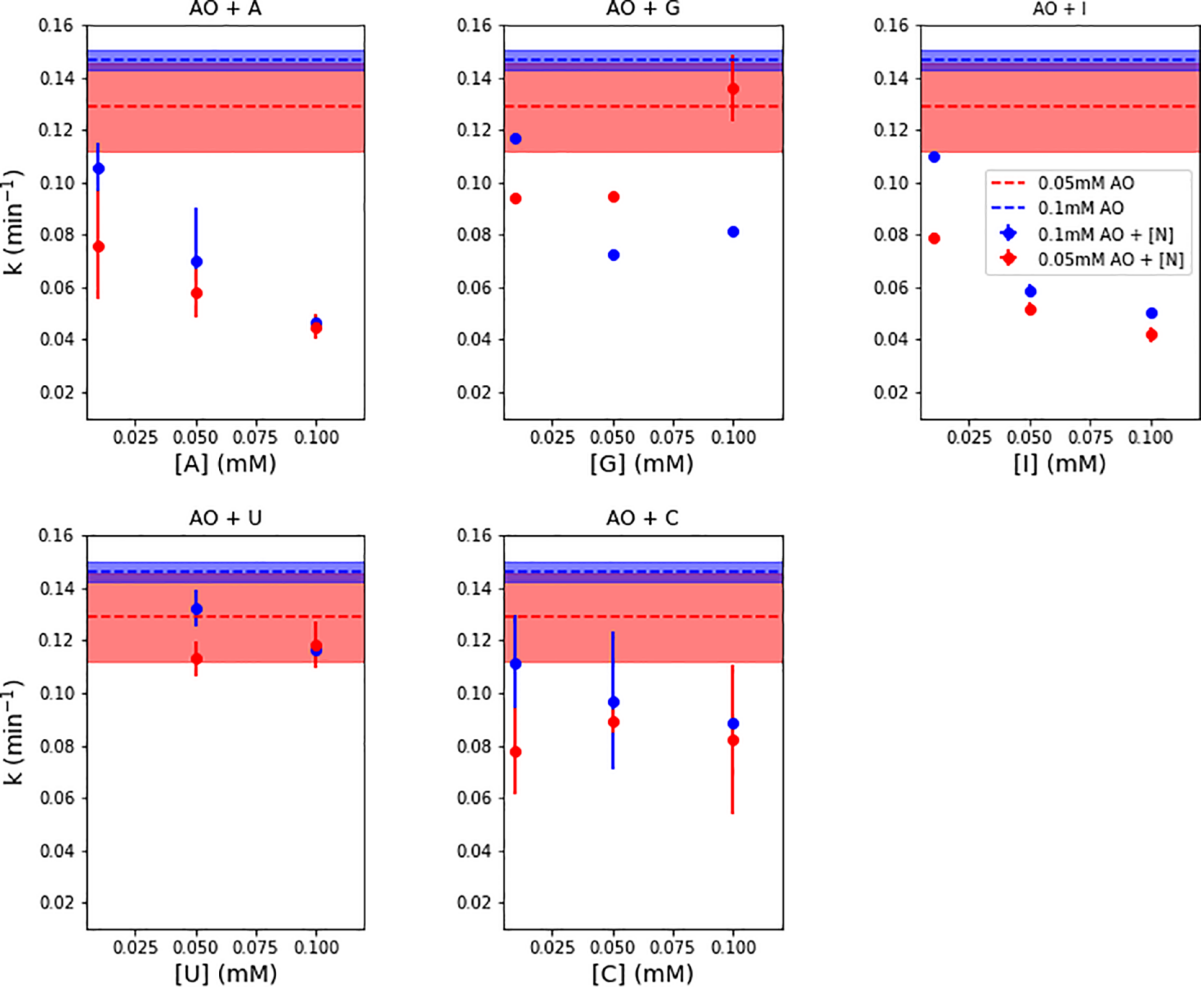
Rate constant for AO photodestruction alone (shaded regions) and with addition of various concentrations of nucleosides (points). AO was tested in two concentrations (0.05 and 0.1 mM, red and blue, respectively). Nucleosides were added in concentrations of 0.01, 0.05, and 0.1 mM. We find that the addition of nucleosides can decrease the rate of photodestruction of AO. The purines were better at shielding AO, with the best protection provided by A. The pyrimidines may have marginally decreased the rate of AO photodestruction, but U was less efficient than C.

Now equipped with methods for determining the concentrations of AO with irradiation time for both purine and pyrimidine co-irradiation experiments, we then determined the rate constants for AO destruction. Figure 6 shows these rate constants for two different concentrations of AO, with and without the addition of nucleosides in varying concentrations. The rate constant for AO degradation alone at concentrations of 0.05 and 0.1 mM is shown in shaded regions (red and blue, respectively), where the variation in rate constant indicates the error from the standard deviation of the duplicate set. The degradation rate constant for 0.05 mM AO and 0.1 mM AO when co-irradiated with varying concentrations of different nucleosides is shown by the red and blue points, respectively. We find that the presence of nucleosides decreases the degradation rate of AO to varying degrees, depending on the identity and concentration of the nucleoside. Generally, higher concentrations of the nucleoside decrease the rate of photodestruction, as would be expected—the increased amount of nucleoside absorbs more UV light and has a stronger shielding effect for AO destruction by UV. The purine nucleosides (A, G, and I) show better protection capabilities, yielding half-life enhancements of roughly a factor of 2 at the higher concentrations. The pyrimidine nucleosides are less effective at protecting AO: C shows marginal half-life enhancements, but the effect of U is not as significant. This is perhaps not surprising given that the pyrimidines are also susceptible to their own UV photodamage by photohydrate formation. Once pyrimidine photohydrates are formed, they absorb less UV light, and therefore do not provide as effective a shield for AO. It is worth pointing out that we find roughly the same amount of protection from the addition of 0.1 mM purine nucleosides as the self-shielding of 1 mM AO. So, production of nucleosides does not have to reach as high concentrations as AO production in order to offer the same protective effects. Therefore, moderate concentrations of nucleosides could act as a UV shield to allow this borderline-photounstable molecule to survive longer.

**Figure 7:**
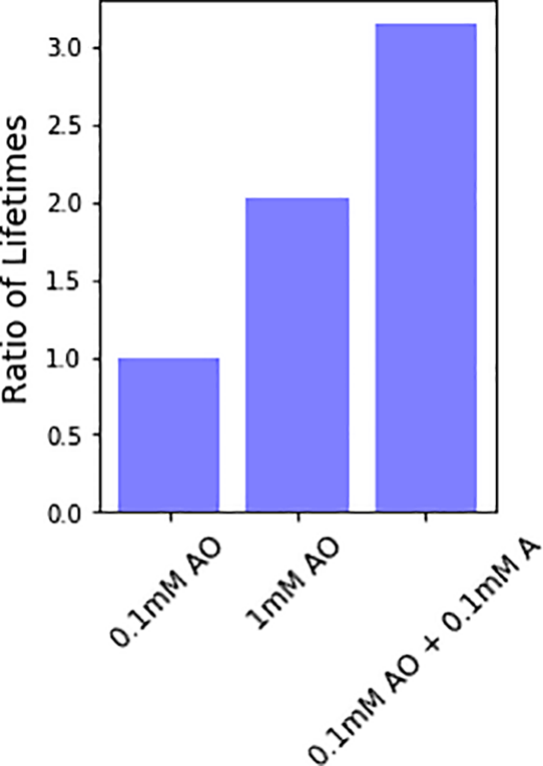
Ratio of lifetimes of 1 mM AO or 0.1 mM AO + 0.1 mM adenosine, relative to the lifetime of 0.1 mM AO. This is a unitless ratio showing the enhancement of lifetime of AO at different concentrations or in the presence of additives, as specified. The lifetime of AO to UV light (as compared to 0.1 mM AO alone) is enhanced by a factor of 3 and 2 for the addition of 0.1 mM A and for 1 mM AO, respectively.

Figure 7 shows the ratio of lifetimes of 1 mM AO and 0.1 mM AO + 0.1 mM adenosine, relative to the lifetime of 0.1 mM AO (also shown). This ratio is a unitless comparison of the enhancement of lifetime of AO at higher concentrations or with additives, with respect to the lifetime of 0.1 mM AO, as specified. We note that we previously determined the lifetime of 0.1 mM AO under solar-like irradiation conditions on the early Earth to be roughly 7 h.^30^ Modest (e.g., 2–3×) enhancements in lifetime are seen with the addition of the nucleoside or when AO is present in higher concentrations such that it self-shields.

While UV light can damage biomolecules such as nucleosides and intermediates such as AO, some prebiotic chemistry uses UV light as a source of energy for driving prebiotic chemistry, as is the case in the Sutherland network (e.g., Xu et al.,^27^ Patel et al.^5^). Therefore, some balance between productive synthetic photochemistry and destructive photodegradation must be reached if the prebiotic network is to be considered plausible. As prebiotic synthetic reactions occur and produce UV-absorbing intermediates and products, less UV light is available to drive synthetic reactions; however, before these products are made, access to UV light to drive synthesis should not be an issue. There could be a point when the synthesis becomes self-terminating, when the products of prebiotic synthesis absorb significant amounts of UV, effectively cutting off the continued synthesis.

**Figure 8:**
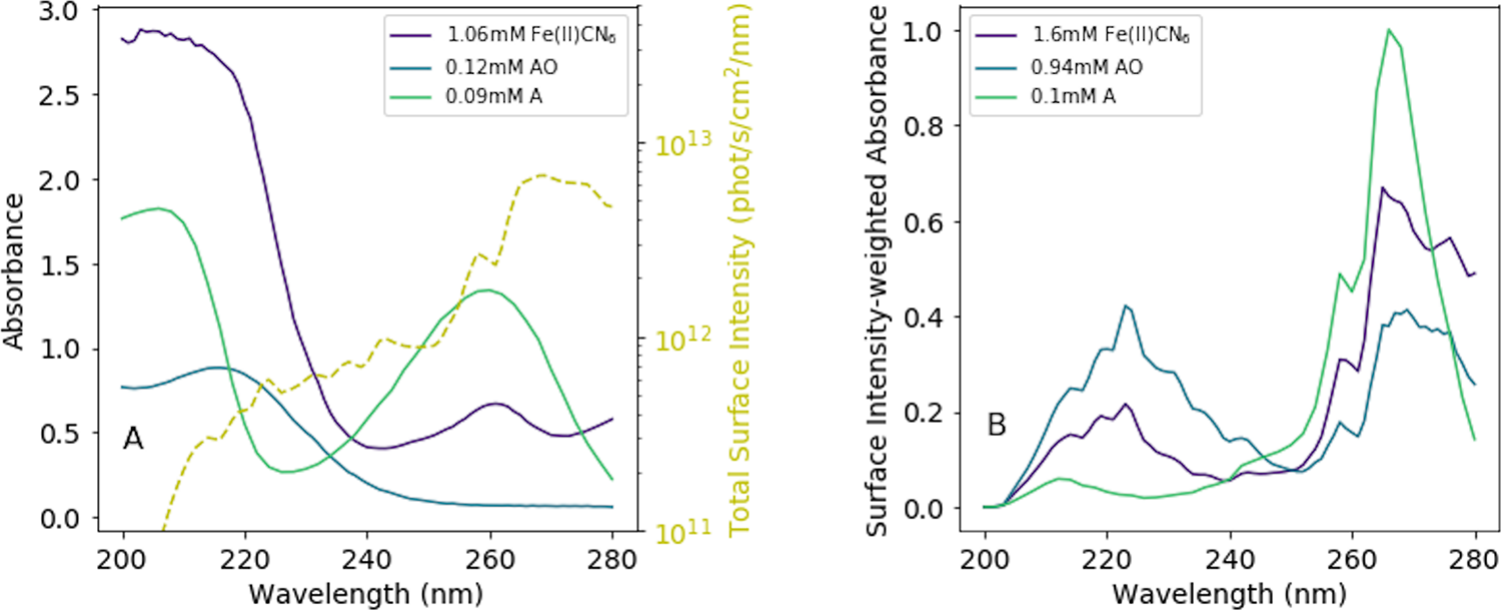
(A) Absorption spectra of Fe(II)CN_6_, AO, and adenosine measured in the laboratory (solid lines) and calculated total surface intensity on the early Earth, from Ranjan and Sasselov.^12^ (B) Total surface intensity-weighted absorbance of Fe(II)CN_6_, AO, and A, in concentrations such that each molecule absorbs equal amounts of UV light when integrated from 200 to 280 nm.

To better understand this issue, we can ask the following: at what ratios of reactants, intermediates, and products will each absorb equal amounts of UV light present on the surface of the early Earth? As an example, we use ferrocyanide (Fe(II)CN_6_) as a reactant, AO as an intermediate, and adenosine as a product, though we note that more complex mixtures are likely. In this simple model, the absorption of UV light by ferrocyanide can be viewed as positive since this will drive the synthesis of precursor molecules. Alternatively, UV absorption by AO and by adenosine could lead to photodegradation of these molecules, which could have a negative effect on prebiotic chemistry. The absorption spectra of the three molecules were measured in the laboratory (Figure 8A, solid lines). Ranjan and Sasselov^12^ simulated the solar UV intensity on the surface of the early Earth using a two-stream radiative transfer model, shown as the dashed line in Figure 8A. By taking the product of the absorption spectra and the surface intensity, we obtain the surface intensity-weighted absorbance of each sample as a function of wavelength (Figure 8B), which is then integrated from 200 to 280 nm to obtain the total absorbance of each sample, weighted by the surface intensity of UV light. If we assume a concentration of 0.1 mM adenosine, we can then calculate the concentrations of ferrocyanide and 2-aminooxazole that would have equal total absorbance to adenosine. We find that 1.6 mM Fe(II)CN_6_, 0.94 mM AO, and 0.1 mM A have equal integrated absorbance from 200 to 280 nm, when weighted by the surface intensity. In order for the total light absorbed between 200 and 280 nm to be equal, the ratio of Fe(II)CN_6_ to AO to adenosine must be 16:9.4:1, respectively.

The above calculation should be viewed as a toy model; there are numerous other molecules and complications that could be considered, but we present this model to show that the weighted UV absorbance of these three molecules does not differ by orders of magnitude. Consequently, if ferrocyanide is present in concentrations larger than adenosine by a factor of >16, ferrocyanide will absorb most of the UV light, allowing UV-driven synthesis to still occur. However, once the synthesis produces similar concentrations of intermediates and products as the reactant ferrocyanide, most of the light will be absorbed by the intermediates and products, probably leading to a reduction in the UV-driven prebiotic synthesis and a switch to increased UV-driven degradation of intermediates and products. We expect that the system would ultimately approach a steady state, where the rates of synthesis and degradation are balanced. Ultimately, to attain a plausible continuous path toward the origins of life, the synthesis of molecules with increasing complexity (i.e., AO and adenosine) needs to be linked to the synthesis of oligomers and polymers of genetic material (e.g., RNA in the RNA World hypothesis) and finally to self-replicating protocells.

## Conclusions

We have used AO as an example of a UV-sensitive molecule in this study. AO plays a key role as an intermediate toward building up the pyrimidine ribonucleotides and was found to be the least photostable of three 2-aminoazoles, with a half-life of 7 h under the UV environment on the early Earth.^30^ While we use AO as an example and find the results for protection and half-life enhancement for this molecule, the shielding mechanisms investigated here should be robust across a variety of molecules. Our results for AO imply that UV photounstable molecules may find their lifetimes enhanced by being present in increasing concentrations or from the presence of other UV-absorbing molecules in solution, such as ribonucleosides. The effectiveness of these protection mechanisms depends on the concentrations of molecules used. The degree of shielding needed may vary from molecule to molecule, depending on its inherent UV photostability and the rates at which it is being produced. The balance between UV-driven synthetic chemistry and the UV-driven photodegradation of intermediates and products must also be considered. We find that the balance between synthetic and destructive chemistry could allow both to occur, but we recognize that this is a potential issue and could place some relevant constraints on prebiotic networks making use of UV light to drive synthesis. We encourage continued consideration of these issues as various prebiotic networks are further elucidated and additional constraints on concentrations and reaction timescales are determined. In particular, determination of the reaction rates of specific steps in synthetic pathways, as well as their dependence on environmental factors including pH, temperature, and concentration, will enable better modeling of plausible planetary scenarios, including effects such as day/night cycles, evaporation, etc. By continuing to examine the conditions for plausible reaction networks, including UV-driven synthesis and destruction and the various shielding mechanisms studied here, we may be able to enhance our understanding of prebiotic chemistry on the early Earth and place valuable constraints and limitations on reasonable scenarios for origins of life chemistry to occur.

## Experimental Section

### Self-Shielding

AO was irradiated spectrosil quartz cuvettes (Starna Cells part number 9-Q-10-GL14-C) in varying concentrations (0.1, 0.5, 1, 5, 10, and 60 mM) in a RPR-200 reactor, with mercury emission lamps (254 nm) for times varying from 10 min to 4 h. During the irradiation, small aliquots of the solution were removed and diluted such that the initial concentration of AO would have been 0.1 mM. The UV–Vis absorption spectra of the diluted solutions were measured from 200 to 350 nm using an Amhersham Sciences Ultrospec 3100 pro spectrophotometer to determine the concentration of AO as a function of time throughout the irradiation. The logarithm of the concentration plotted against time gives a straight line, the slope of which represents the rate constant of the reaction. We determined the rate constants for each initial concentration of AO. Experiments were repeated in duplicate to obtain an average and error for the rate constants.

### Nucleoside Co-irradiation

Solutions of AO (0.05 or 0.1 mM) were made with varying concentrations (0.01, 0.05, or 0.1 mM) of different nucleosides (A, G, C, U, and I) and irradiated at 254 nm (RPR-200 reactor, mercury emission lamps) in spectrosil quartz cuvettes (Starna Cells part number 9-Q-10-GL14-C). The UV–Vis absorption spectra were measured from 200 to 350 nm using an Amhersham Sciences Ultrospec 3100 pro spectrophotometer throughout the course of the irradiations (lasting from 10 to 30 min). The UV spectra were used to determine the concentration of AO over the course of the irradiation, which was then used to calculate the rate constant (see SI section 4). Experiments were repeated in duplicate to obtain an average rate constant and the associated error.

## Supporting Information Available

General methods, standard curves and extinction coefficients, self-shielding experiments analysis, and nucleoside co-irradiation experiments analysis (PDF)
