# Supplementary material for: Shielding from UV Photodamage: Implications for Surficial Origins of Life Chemistry on the Early Earth
Source: ACS Earth Space Chem. 2021 Jan 29;5(2):239–46. doi: 10.1021/acsearthspacechem.0c00270 (PMC9616438; doi:10.1021/acsearthspacechem.0c00270)
Supplement: Supplementary file 1 — sp0c00270_si_001.pdf [file sp0c00270_si_001.pdf]

Supporting Information

Shielding from UV photodamage: implications for surficial origins of life chemistry on the early Earth

Zoe R. Todd, Jack W. Szostak, Dimitar D. Sasselov

Table of Contents

1. General Methods
2. Standard Curves and Extinction Coefficients
3. Self-shielding experiments analysis
4. Nucleoside co-irradiation experiments analysis

1. General Methods

2-aminooxazole (97%) was purchased from CombiBlocks. Adenosine (>99%), uridine (>99%), cytidine (>99%), guanosine (>98%), and inosine (>99%) were purchased from Sigma Aldrich. During irradiation experiments, solutions of the appropriate concentration of AO was prepared in deionized water. During the nucleoside co-irradiation experiments, the appropriate concentration of nucleoside was added to the solution. The samples were transferred to a Spectrosol quartz cuvette with a screw top (Starna Cells part number 9-Q-10-GL14-C). Initial UV absorption spectra was taken before irradiation (200-350 nm). The samples were irradiated in a Rayonet RPR-200 reactor using 8 mercury lamps (primary emission at 254 nm, Figure S1). At periodic intervals throughout the irradiation, the sample was removed for analysis. In nucleoside co-irradiation experiments, a UV absorption spectrum was taken at each timepoint. In self-shielding experiments, an aliquot of the solution was removed and diluted by the appropriate amount such that the initial concentration of AO would have been 0.1mM. This diluted sample was then measured by UV absorption spectroscopy.

Figure S1. The Rayonet reactor (RPR-200) houses up to 16 lamps total, 8 of which were used in experiments in this paper. Mercury lamps, with primary emission at 254 nm, were used for irradiations. The power from 8 lamps total was measured to be 5.2±0.4 mW.

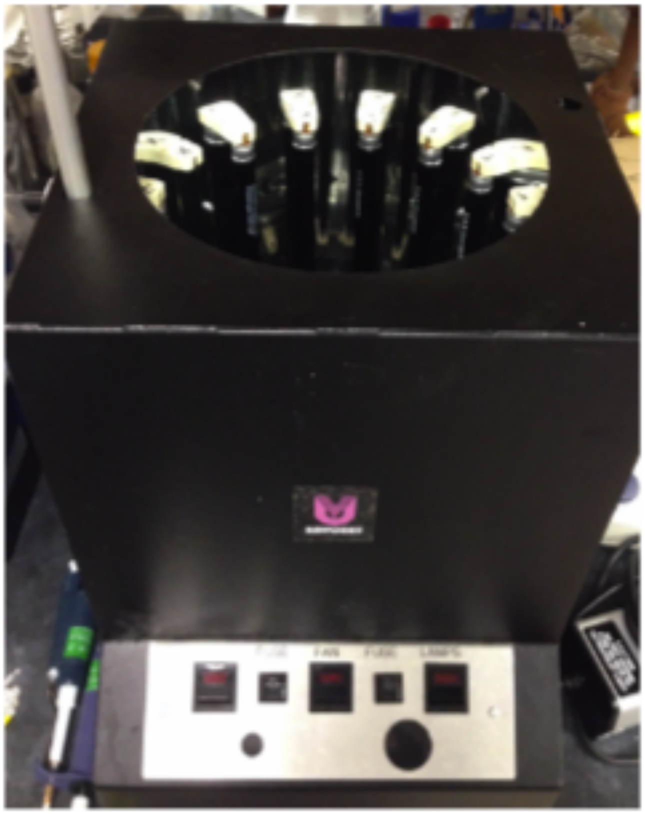

2. Standard Curves

In order to relate the UV absorption signal of AO to its concentration, we generated standard curves of AO. Figure S2 shows the absorbance spectra of AO at varying concentration (panel A) and the absorption at the peak wavelength (215 nm) as a function of concentration (panel B). The equation relating the peak absorption to concentration for AO is:

$$conc_{AO} = 0.153A_{AO,215nm} - 0.0162 \qquad (1)$$

To determine the concentrations of nucleosides for the extraction procedures, we measured the UV absorption spectra of the relevant nucleosides. We used known extinction coefficients at 260 nm from the literature for these nucleosides to determine the precise concentration of the sample. Once the concentration was known, the extinction coefficient at 215 nm, the wavelength necessary for concentration extraction with AO, was calculated:

$$\epsilon_{215nm,A/I/G} = \frac{A_{215nm,A/I/G}}{c_{215nm,A/I/G}l} \qquad (2)$$

Figure S2. (A) Absorption spectra of varying concentrations of AO. (B) Absorbance at 215 nm vs. concentration shows a linear trend, with a fit given by equation (1). This fit enables the concentration of AO to be calculated from the absorption spectrum of a sample.

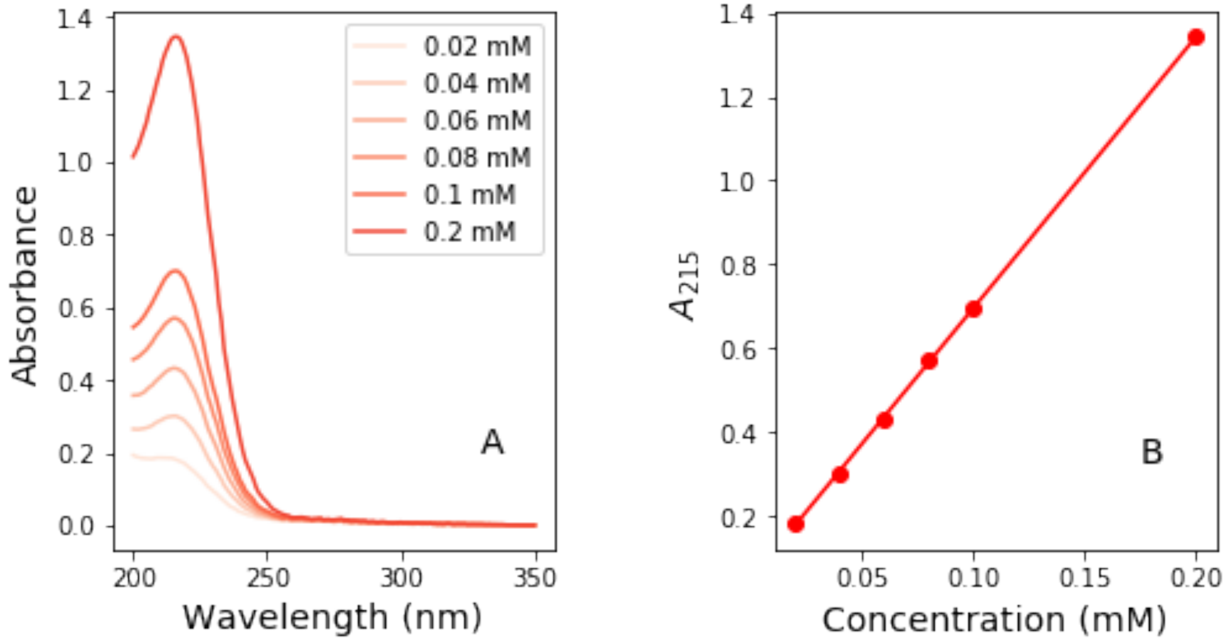

The extinction coefficients for the purine nucleosides and AO at 215 and 260 nm are shown in Table A1.

Table A1. Extinction coefficients for AO and the purine nucleosides, determined from the literature and experiments measuring the UV absorption spectra.

**Sample**	$\epsilon_{215\ nm}$ **(mM^{-1} cm^{-1})**	$\epsilon_{260\ nm}$ **(mM^{-1} cm^{-1})**
AO	7.48	0.514
Adenosine	12.7	15.4
Inosine	6.68	7.93
Guanosine	5.92	12.1

3. Self-shielding experiments analysis

For self-shielding experiments, we irradiated varying concentrations of AO solutions, from 0.1-60 mM, in the Rayonet RPR-200 (Figure S1), with primary emission at 254 nm. To monitor the progress of the reaction, an aliquot of the solution was removed and diluted such than the initial concentration of AO would have been 0.1mM. So, for example, the 0.1mM solution was not diluted and was just measured by UV spectroscopy throughout the irradiation, while the 0.5mM AO solution was diluted 5x, 1mM by 10x, etc. The concentration of AO was determined by UV spectroscopy, then corrected for the dilution factor to give the concentration of the solution undergoing irradiation.

Rate constants were determined by plotting the logarithm of AO concentration vs. irradiation time, which gave a linear trend as expected for a first order reaction. The slope of the fit is the rate constant of the reaction; rate constants were calculated for each initial concentration of AO. All experiments were repeated in duplicate; the points in Figure 2 represent the average and the error bars show the standard deviation.

The irradiations for self-shielding experiments varied from 15 minutes to 4 hours, depending on the initial concentration of AO. Higher concentrations were irradiated for longer time periods, since the photodestruction of AO required longer irradiation times.

4. Nucleoside co-irradiation experiments analysis

During nucleoside co-irradiation experiments, two different concentrations of AO (0.05 and 0.1 mM) were tested with varying identities and concentrations of nucleosides. We tested five nucleosides: adenosine, uridine, cytidine, guanosine, and inosine; the concentrations of nucleoside were 0.01, 0.05, and 0.1mM. The appropriate concentration of AO and nucleoside were prepared in 1 mL deionized water and irradiated in the RPR-200 reactor (emission at 254 nm) for 12-30 minutes. At given timepoints during the irradiation, the cuvette was removed and measured by UV absorption spectroscopy. The analysis and determination of the concentration of AO, and therefore degradation rate, was different for the purine and pyrimidine ribonucleosides.

The purine ribonucleosides have a maximum absorption feature near 260 nm, which is observed to decrease upon UV irradiation without the introduction of new UV absorption features. This enables the extraction and calculation of the concentrations of both AO and the purine nucleoside, from solving a system of two equations:

$$A_{215nm} = \epsilon_{AO,215} c_{AO} l + \epsilon_{A/I/G,215} c_{A/I/G} l \qquad (3)$$

$$A_{260nm} = \epsilon_{AO,260} c_{AO} l + \epsilon_{A/I/G,260} c_{A/I/G} l, \qquad (4)$$

where ϵ are the extinction coefficients, determined from the standard curves in section 2. Solving the system of equations at each timepoint allows the concentration of AO and the purine nucleoside to be calculated during irradiation. These concentrations are then fit using first order kinetics to determine the rate constant of degradation for AO and the purine. AO is much more photounstable than the purines, despite the primary emission of irradiation aligning much better in wavelength with the absorption feature of the ribonucleoside, showing that purine ribonucleosides are quite photostable, while AO is not.

The pyrimidine ribonucleosides, while still showing a decrease in signal near their absorption peak at 260 nm upon UV irradiation, show increasing absorption features at shorter

wavelengths (~<240 nm for cytidine ~<220 nm for uridine). Since this changing absorption due to the pyrimidines overlaps with the absorption maximum of AO, the simple concentration extraction used for the purine nucleosides could not be performed. Instead, we obtained rough estimates for the degradation of AO during co-irradiation with pyrimidines by performing an additional irradiation with only the pyrimidine ribonucleoside in the appropriate concentrations. The pyrimidine-alone irradiations were monitored at precisely the same timepoints as the co-irradiation experiments. The spectra of the pyrimidine-alone irradiation at a given timepoint were subtracted from the pyrimidine+AO co-irradiation spectra to obtain difference spectra where the absorption changes should primarily be due to changing concentrations of AO. However, we note that this method is not as ideal and may only provide estimated concentrations of AO throughout these co-irradiations. We find that pyrimidine nucleosides are less effective at protecting AO, but also suggest using caution when interpreting the pyrimidine co-irradiation experiments since this procedure of subtracting spectra that could in theory introduce artifacts. We however note that when this subtraction method is used for the purine nucleoside co-irradiation experiments, similar AO destruction rates are recovered compared to the concentration extraction method, supporting the validity of this procedure.
